# Increased FIO_2_ influences SvO_2_ interpretation and accuracy of Fick-based cardiac output assessment in cardiac surgery patients: A prospective randomized study

**DOI:** 10.1097/MD.0000000000027020

**Published:** 2021-09-10

**Authors:** Sheng-Yi Lin, Feng-Cheng Chang, Jr-Rung Lin, An-Hsun Chou, Yung-Fong Tsai, Chia-Chih Liao, Hsin-I. Tsai, Chun-Yu Chen

**Affiliations:** a Department of Anesthesiology, Chang Gung Memorial Hospital, Linkou Medical Center, Taoyuan, Taiwan; b College of Medicine, Taipei Medical University, Taipei, Taiwan; c College of Medicine, Fu Jen Catholic University, Taipei, Taiwan; d Clinical Informatics and Medical Statistics Research Center and Graduate Institute of Clinical Medicine, Chang Gung University, Taoyuan, Taiwan; e Biostatistics, National Taiwan University, Taipei, Taiwan; f College of Medicine, Chang Gung University, Taoyuan, Taiwan.

**Keywords:** cardiac output, Fick principle, fraction of inspired oxygen, mixed venous oxygen saturation

## Abstract

**Introduction:**

The study aimed to reveal how the fraction of inspired oxygen (FIO_2_) affected the value of mixed venous oxygen saturation (SvO_2_) and the accuracy of Fick-equation-based cardiac output (Fick-CO).

**Methods:**

Forty two adult patients who underwent elective cardiac surgery were enrolled and randomly divided into 2 groups: FIO_2_ < 0.7 or >0.85. Under stable general anesthesia, thermodilution-derived cardiac output (TD-CO), SvO_2_, venous partial pressure of oxygen, hemoglobin, arterial oxygen saturation, arterial partial pressure of oxygen, and blood pH levels were recorded before surgical incision.

**Results:**

Significant differences in FIO_2_ values were observed between the 2 groups (0.56 ± 0.08 in the <70% group and 0.92 ± 0.03 in the >0.85 group; *P* < .001). The increasing FIO_2_ values lead to increases in SvO_2_, venous partial pressure of oxygen, and arterial partial pressure of oxygen, with little effects on cardiac output and hemoglobin levels. When comparing to TD-CO, the calculated Fick-CO in both groups had moderate Pearson correlations and similar linear regression results. Although the FIO_2_ <0.7 group presented a less mean bias and a smaller limits of agreement, neither group met the percentage error criteria of <30% in Bland-Altman analysis.

**Conclusion:**

Increased FIO_2_ may influence the interpretation of SvO_2_ and the exacerbation of Fick-CO estimation, which could affect clinical management.

**Trial Registration:**

ClinicalTrials.gov ID number: NCT04265924, retrospectively registered (Date of registration: February 9, 2020).

## 1 Introduction

Optimizing stroke volume and cardiac output (CO) can improve the outcome for patients undergoing major surgeries. The standard approach to estimating CO levels involves the use of a pulmonary artery catheter in conjunction with thermodilution based on the Stewart-Hamilton equation.^[1]^ However, the invasiveness and inherent risks of this approach^[2]^ have led to the development of alternative methods to measure CO levels.

The calculated cardiac output based on Fick principle (Fick-CO) has been widely adopted in catheterization and pediatric cardiology, particularly for patients with congenital heart diseases.^[3]^ The Fick principle is based on the observation that total oxygen consumption by the body (V’O_2_) is equal to the difference between the amount of oxygen leaving and returning to the lung. Note that pulmonary oxygen consumption is negligible.^[4]^

A number of researchers have reported a strong correlation between thermodilution-derived cardiac output (TD-CO) and Fick-CO,^[5,6]^ while others have reserved comment on this issue.^[7–9]^ To further investigate the discrepancy, however, there has been relatively little research on the influence of the variables related to oxygen content in the Fick equation–such as arterial partial pressure of oxygen (PaO_2_), mixed venous oxygen saturation (SvO_2_), and venous partial pressure of oxygen (PvO_2_). These variables could be affected by the fraction of inspired oxygen (FIO_2_), manifesting as variations in Fick-CO levels. Furthermore, the value of SvO_2_ is also frequently used as an indicator of tissue perfusion adequacy. If different FIO_2_ leads to different SvO_2_ values, the clinical management may be affected. Therefore, in this study, we sought to clarify the influence of FIO_2_ levels on SvO_2_ and other variables in the Fick equation in patients undergoing cardiac surgery. We then assessed the influence of each variable on the accuracy of Fick-CO measurements.

## 2 Methods

### 2.1 Study design

This prospective randomized study was approved by the Institutional Review Board of Chang Gung Memorial Hospital in Taiwan (registration number: 104-7177B) and was retrospectively registered with ClinicalTrials.gov (ID number: NCT04265924, date of registration: February 9, 2020). At the time of conducting this study, the contemporary trend favored high intraoperative FIO_2_ to prevent surgical site infection; World Health Organization guidelines published in November 2016 further advocated 80% FIO_2_ for surgical patients undergoing general anesthesia with tracheal intubation.^[10]^ Hence, we assigned our patients randomly into 2 groups: the higher FIO_2_ group (FIO_2_ > 0.85) and the lower FIO_2_ group (FIO_2_ < 0.7). According to our past observation and the results from previous relevant studies,^[11,12]^ with the assumption of a 5% difference of SvO_2_ between the 2 groups in our study, the number of subjects required was 20 in each group (power =80% and α = 0.05). In case of drop-outs, the determined target sample size was 50.

### 2.2 Participants

Adult patients (age ≥20 years) who underwent elective cardiac surgery and provided signed informed consent were included. Any patients with an intra-cardiac shunt were excluded.

### 2.3 Randomization

The enrolled patients were allocated randomly with a 1:1 ration into the higher FIO_2_ group (FIO_2_ > 0.85) or the lower FIO_2_ group (FIO_2_ < 0.7). Randomization was performed according to a computer-generated randomization number list, from 1 to 50, which was created before commencing the study by an independent statistician not involved in data analysis. In the time order of enrollment, each patient got his corresponding number on the list; odd number represented the higher FIO_2_ group (FIO_2_ > 0.85), while even number was the lower FIO_2_ group (FIO_2_ < 0.7).

### 2.4 Interventions

The drug selection and dosage used for anesthetic induction varied basing on clinical conditions. After intubation, FIO_2_ was adjusted based on the assigned group. General anesthesia was maintained using sevoflurane (1.5%–2.5%), fentanyl (0.5–2 μg/kg according to the clinical condition), and cisatracurium (2–4 mg/30 minutes). All patients were mechanically ventilated at a tidal volume of 8 to 10 mL/kg at a respiratory rate of 8 to 14 per minute to maintain end-tidal CO_2_ concentrations of 35 to 45 mm Hg. Throughout the study, the oximeter values were maintained at ≥98%.

### 2.5 Data collection

For every patient, a pulmonary artery catheter was inserted into the internal jugular vein, and the position of the tip was confirmed by pressure waves and transesophageal echocardiography; then it was connected to a Vigilance II Monitor (Edwards Lifesciences, CA, USA) or an Abbott Q2 Plus CCO/ SvO_2_ Computer (Abbott Laboratories, IL, USA) to obtain continuous measurements of TD-CO levels. Blood samples drawn from the pulmonary artery catheter were used to monitor SvO_2_ and PvO_2_ levels. An arterial pressure catheter was inserted into the radial artery to allow analysis of hemoglobin (Hb), arterial oxygen saturation, and PaO_2_ levels. All arterial and venous gas concentrations were derived using a NOVA Critical Care Xpress Blood Gas Analyzer (Nova Biomedical, Waltham, MA, USA), which was calibrated daily according to the manufacturer's instructions, and with regular maintenance every 3 months to ensure accuracy. Under stable general anesthesia with assigned FIO_2_ for 30 minutes, data pertaining to TD-CO and blood analysis (Hb, arterial oxygen saturation, SvO_2_, PaO_2_, and PvO_2_) were recorded prior to surgical incision. Meanwhile, we also recorded body temperature and pH values of arterial blood in consideration of their influences on V’O_2_ and O_2_-Hb dissociation.

### 2.6 Fick equation

Fick-based cardiac output is the ratio of V’O_2_ to the difference between arterial (CaO_2_) and venous (CvO_2_) oxygen content, as follows:

$$\begin{array}{l} CO\left(\frac{L}{\min}\right)=\frac{V'O_{2}}{CaO_{2}-CvO_{2}}\\ =\frac{V'O_{2}(ml/\min)}{1.34*Hb\left(\frac{g}{dL}\right)*(SaO_{2}-SvO_{2})(\%)*10+0.03*(PaO_{2}-PvO_{2})(mmHg)} \end{array}$$

The standard approach to obtaining CO levels is the direct Fick method; however, this is impractical in a clinical setting due to its complexity to obtain V’O_2_ measurements directly. In addition, when FIO_2_ exceeds 0.6, measurements of V’O_2_ tend to be inaccurate.^[13,14]^ Thus, we adopted the indirect Fick method. The estimated V’O_2_ values were obtained from the LaFarge equation^[15]^ as below:

$$\begin{array}{l} Male:V'O_{2}(ml/\min/m^{2})=138.1-11.49*\log\;age\;(years)+0.378*HR\;(beats/\min)*BSA(m^{2})\\ Female:V'O_{2}(ml/\min/m^{2})=138.1-17.04*\log\;age\;(years)+0.378*HR\;(beats/\min)*BSA(m^{2}) \end{array}$$

In the above equation, HR stands for heart rate, and BSA is body surface area.

### 2.7 Statistical analysis

All statistical analysis was performed using SPSS (version 22.0, SPSS Inc., Chicago, IL). A paired *t*-test was used to determine the statistical significance of differences between 2 independent sets of continuous variables. The Pearson correlation coefficient and simple linear regression analysis were used to evaluate the correlation between Fick-CO and TD-CO. The degree of agreement and bias between the Fick-CO and TD-CO were evaluated using Bland–Altman analysis corrected for repeated measures.^[16]^ Percentage errors were calculated as 1.96 times the standard deviation of the bias divided by the mean CO of the reference method (TD-CO). A percentage error of <30% was considered acceptable.^[17]^ For all statistical analysis, *P* < .05 was considered statistically significant.

## 3 Results

**Figure 1:**
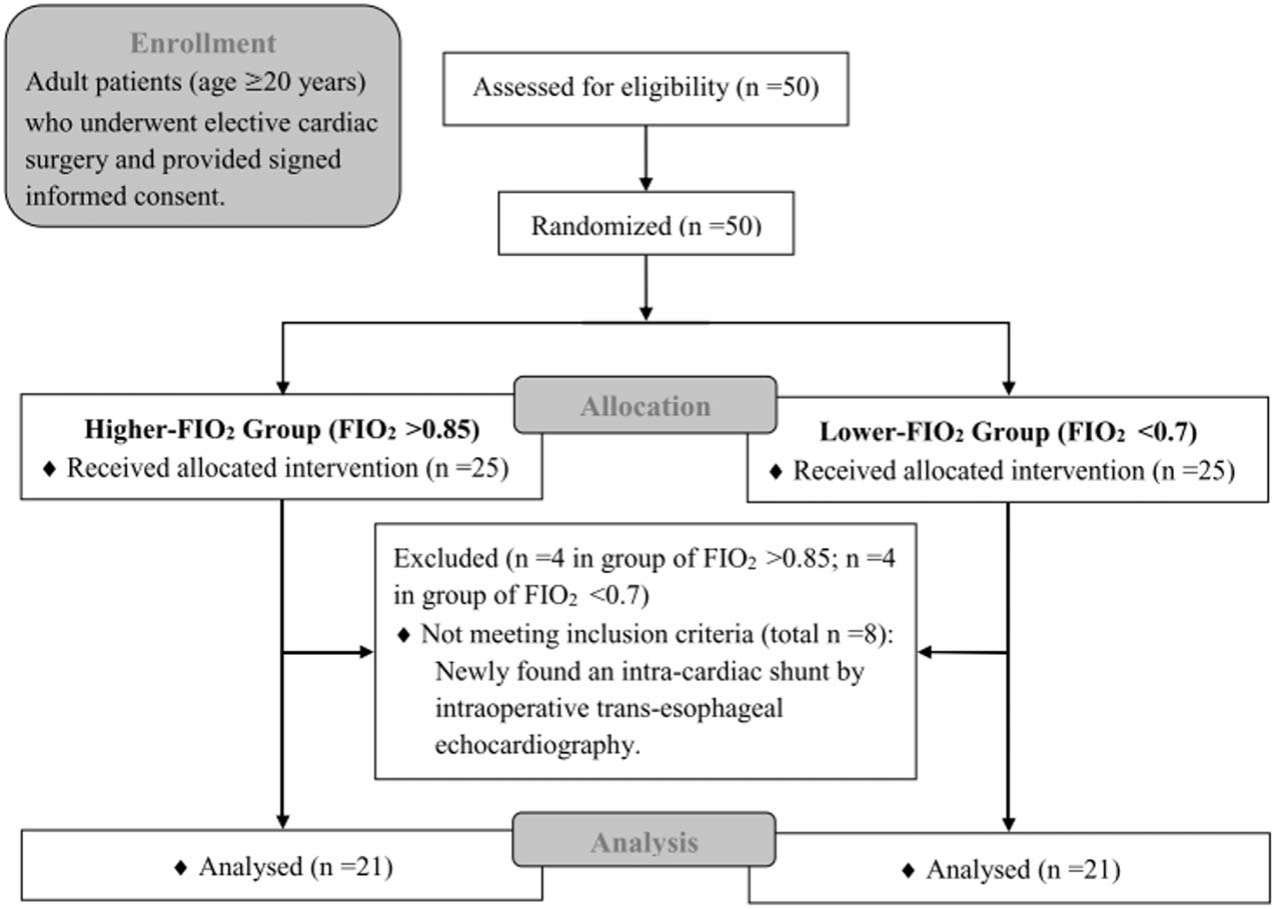
Study Flow Chart.

Fifty patients were enrolled between December 2015 and June 2016. Eight of them were excluded due to an intra-cardiac shunt newly found by trans-esophageal echocardiography during the surgeries (Fig. 1). A total of 42 patients underwent final analysis. Patient characteristics are listed in Table 1. Each of the groups (FIO_2_ > 0.85 and FIO_2_ < 0.7) included 21 patients. Fick-CO and TD-CO levels were recorded prior to incision. The TD-CO values in the two groups were similar (Table 1). During the study period, none of the patients needed inotropes.

**Table 1:** Patient characteristics.

	Higher-FIO_2_ group (FIO_2_ > 0.85, n = 21)	Lower-FIO_2_ group (FIO_2_ < 0.7, n = 21)
Preoperative data		
Age (yrs)	60 ± 13	63 ± 13
Gender (M/F)	12/9	16/5
Ejection fraction (%)	65 ± 13	56 ± 17
Pulmonary Hypertension: Moderate/Severe	3/0	4/1
Ventilatory impairment in pulmonary function test: moderate/severe	4/1	6/1
HTN	10	10
DM	8	11
CVA history	2	3
ESRD	2	2
Operation		
CABG	9	11
Valve	10	10
Aortic Root	2	0
TD-CO	3.6 ± 1.5	3.6 ± 0.6

### 3.1 Effects of fraction of inspired oxygen on mixed venous oxygen saturation, arterial partial pressure of oxygen, and venous partial pressure of oxygen

Significant differences in FIO_2_ values were observed between the 2 groups (0.92 ± 0.03 in the >0.85 group and 0.56 ± 0.08 in the <0.7 group; *P* < .001); however, no significant differences in body temperature, pH, V’O_2_, or Hb values were observed (Table 2). SvO_2_, PaO_2_, and PvO_2_ values were significantly higher in the FIO_2_ > 0.85 group, and the difference between PaO_2_ and PvO_2_ was statistically pronounced (Table 2; *P* = .01, *P* < .001, *P* = .01, and *P* < .001, respectively).

**Table 2:** Results of each variables and statistical significance (*P* value) between 2 groups.

Parameter	Higher -FIO_2_ group (FIO_2_ > 0.85, n = 21)	Lower -FIO_2_ group (FIO_2_ < 0.7, n = 21)	*P* value
FIO_2_	0.92 ± 0.03	0.56 ± 0.08	<.001
BT	36.1 ± 0.6	36.2 ± 0.5	.97
pH	7.4 ± 0.0	7.4 ± 0.0	.15
V’O_2_	156.0 ± 13.6	158.8 ± 10.2	.46
Hb	12.3 ± 1.5	11.6 ± 1.1	.11
SvO_2_	85.1 ± 5.6	79.9 ± 6.4	.01
PaO_2_	402.9 ± 71.8	236.4 ± 102.9	<.001
PvO_2_	53.0 ± 7.1	47.3 ± 5.1	.01
PaO_2_ -PvO_2_	349.0 ± 73.0	189.0 ± 100.4	<.001

### 3.2 Effects of fraction of inspired oxygen on the accuracy of Fick-equation-based cardiac output

**Figure 2:**
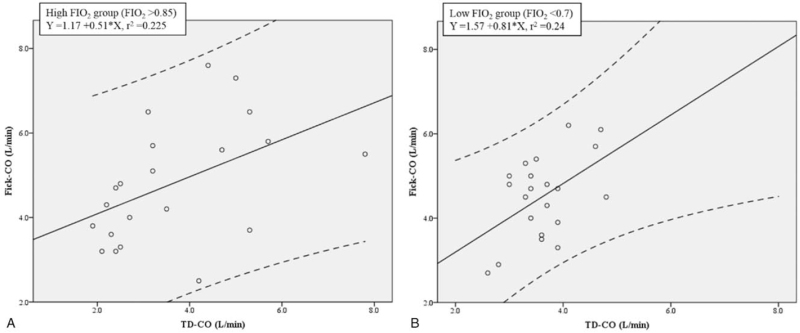
A and B. Simple linear regression between Fick-CO and TD-CO. The continuous line indicates the regression line, and the striped lines are the 95% confidence interval. Each dot represents a patient.

**Figure 3:**
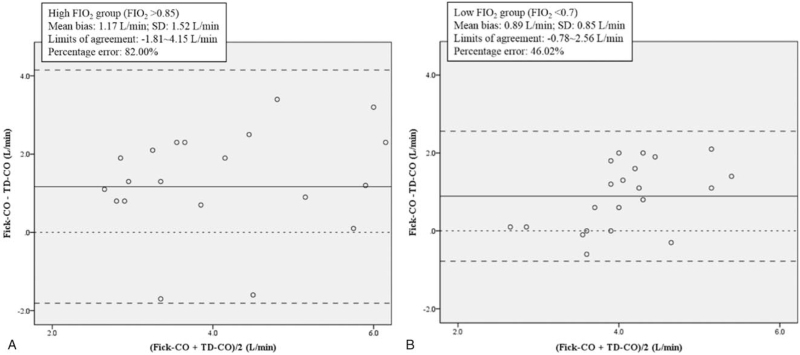
A and B. Bland-Altman Analysis for Fick-CO and TD-CO Measurements. The continuous line indicates the mean bias, the striped lines are the 95% limit of agreement, and the dotted line is the value of 0. Each dot represents a patient.

As indicated by the Pearson correlation coefficient values in Table 3, both of the groups had a moderate correlation to TD-CO, as follows: 0.475 in FIO_2_ > 0.85 group (*P* = .03) and 0.490 in FIO_2_ < 0.7 group (*P* = .02). As shown in Figure 2A and B, both of the groups presented a similar linear regression result between Fick-CO and TD-CO, as follows: FIO_2_ > 0.85 group (*r*^2^ = 0.225) and FIO_2_ < 0.7 group (*r*^2^ = 0.24). Nevertheless, as shown in Figure 3A and B, Bland-Altman analysis revealed a greater discrepancy between Fick-CO measurements and TD-CO values in the FIO_2_ > 0.85 group than in the FIO_2_ < 0.7 group, as follows: FIO_2_ > 0.85 group (mean bias = 1.17; limit of agreement =−1.81∼4.15; and percentage error =82.00%) and FIO_2_ < 0.7 group (mean bias = 0.89; limit of agreement =−0.78∼2.56; and percentage error = 46.02%). Neither group met the percentage error criterion of < 30%.

**Table 3:** Results and statistics of cardiac outputs calculated by Fick method and measured through thermodilution.

	Higher-FIO_2_ group (FIO_2_ > 0.85, n = 21)		Lower-FIO_2_ group (FIO_2_ < 0.7, n = 21)	
	Fick-CO	TD-CO	Fick-CO	TD-CO
Mean ± SD	4.8 ± 1.4	3.6 ± 1.5	4.5 ± 1.0	3.6 ± 0.6
Pearson Correlation (*P* value)	0.475 (.03)		0.490 (.02)	

## 4 Discussion

Our results reveal that increasing FIO_2_ values leads to increases in SvO_2_, PvO_2_, and PaO_2_ as well as a more pronounced difference between PaO_2_ and PvO_2_, with little or no effect on TD-CO and Hb levels. The increase in these values was shown to increase the effects of bias and the percentage error of Fick-CO when using TD-CO as the reference standard. These results indicate the non-negligible influence of FIO_2_ on the clinical interpretation of SvO_2_ and Fick-CO values.

### 4.1 Increased fraction of inspired oxygen vs mixed venous oxygen saturation

Mixed venous oxygen saturation reflects the balance between oxygen consumption and delivery, clinically used as a surrogate for tissue perfusion adequacy. This indicator is sensitive to cardiopulmonary instability. A pronounced decrease in SvO_2_ has been associated with severe impairments in cardiopulmonary circulation,^[18]^ whereas a low and therapy-unresponsive SvO_2_ value is predictive of poor outcomes.^[19,20]^ Thus, many guidelines suggest using SvO_2_ to guide therapy, and a number of researchers suggest maintaining SvO_2_ or central venous saturation above 70% to reduce morbidity and mortality.^[21–23]^ Improvements in SvO_2_ values often indicate the suitability and timeliness of treatments.

Nonetheless, many situations can lead to an increase in SvO_2_ levels, such as hyperdynamic sepsis,^[24]^ intracardiac shunts,^[25]^ liver failure,^[26]^ excessive inotropic administration,^[27]^ and increased carboxyhemoglobin levels.^[26,28]^ Our findings indicate that an increase in FIO_2_ leads to elevated SvO_2_ levels, which is consistent with previous studies.^[11,12,29,30]^ Perry et al reported that each 100 mm Hg increase in PaO_2_ led to a 4.9% increase in SvO_2,_ despite a constant CO.^[30]^ The possible explanation is that hyperoxia may reduce V’O_2_^[31]^ through mechanisms such as a decrease in myocardial oxygen consumption^[32]^ or a reduction in the metabolism of cells and tissues.^[33,34]^ Besides, hyperoxia has been shown to increase arteriolar constriction,^[35,36]^ reducing functional capillary density and nutritive organ blood flow, leading to a subsequent decrease in peripheral oxygen delivery, which can contribute to a reduction in V’O_2_. A reduction in V’O_2_ would tend to increase residual oxygen levels and PvO_2_. Even if there were no changes in oxygen delivery, Perry et al proved that during hyperoxia, there could be an increase in SvO_2_ levels due to an increase in tissue oxygen tension (via the Fickian diffusion of excess dissolved oxygen), resulting in elevated PvO_2_ values.^[30]^ A modest increase in PvO_2_ could lead to a significant increase in SvO_2_ due to the sigmoid shape of the O_2_-Hb dissociation curve.^[12]^ Ultimately, the affected value of SvO_2_ caused by increased FIO_2_ could conceal a situation involving insufficient oxygen delivery, thereby hindering therapy. Therefore, whenever SvO_2_ is used as an indicator to evaluate tissue perfusion or the adequacy of measures aimed at resuscitating critically ill patients,^[21–23,37]^ it is necessary to take FIO_2_ values into account as well as their effect on SvO_2_.

### 4.2 Increased fraction of inspired oxygen vs fick-equation-based cardiac output

An increase in FIO_2_ can affect a number of variables in the Fick-CO equation (SvO_2_, PvO_2_, PaO_2_, and PaO_2_ -PvO_2_), which could influence the accuracy of Fick-CO calculations. In this study, all of these values were significantly higher in the FIO_2_ > 0.85 group than in the FIO_2_ < 0.7 group. Omitting the difference between PaO_2_ and PvO_2_ in the denominator of the fraction, as in a number of previous studies,^[5,6,38]^ would lead to an even greater error in Fick-CO estimation, particularly in cases of hyperoxia.

Notably, in the study of Perry et al— a similar study on swine to evaluate the accuracy of Fick-CO assessments with an increase in FIO_2_,^[30]^ after correction, they reached a conclusion that hyperoxia would not exaggerate the error of Fick-CO, which contradicted our findings. Further analysis of their data revealed that the SvO_2_ values were much lower in swine (58.2 ± 7.27% under FIO_2_ = 0.6, and 61.0 ± 6.7% under FIO_2_ = 0.8), comparing to data in human in our study (79.9 ± 6.4% under FIO_2_ = 0.56, and 85.1 ± 5.6% under FIO_2_ = 0.92). The disparity leads to the different results obtained in the two studies.

One possible reason for the impact of FIO_2_ on the Fick-CO estimation is measurement error. Previous studies have reported that a 10% increase in SvO_2_ would result in an observed erroneous 32.8% increase in Fick-CO estimates, which would become increasingly prominent with a decrease in the arteriovenous difference of oxygen content.^[39]^ This underlines the importance of accounting for the value of FIO_2_ in any research based on Fick-CO. In short, any discussion pertaining to the clinical utility of Fick-CO must take the effects of FIO_2_ into consideration.

### 4.3 Limitations

In this prospective study, we used the LaFarge equation to estimate V’O_2_ instead of measuring V’O_2_ directly, which was the primary limitation of this study. Note that we opted for this approach given the fact that V’O_2_ measurements are prone to inaccuracy when FIO_2_ > 0.6.^[13,14]^ Further study will be required to determine the means by which changes in V’O_2_ alter Fick-CO estimates under elevated FIO_2_ levels.

## 5 Conclusions

An increase in FIO_2_ leads to increases in SvO_2_, PvO_2_, and PaO_2_. As an indicator of tissue perfusion adequacy, this hyperoxia-influencing SvO_2_ value may conceal insufficient oxygen delivery and delay medical treatment. Furthermore, increases in SvO_2_, PvO_2_, and PaO_2_ could exacerbate errors in Fick-CO estimates. Thus, the influence of FIO_2_ should be considered whenever using SvO_2_ and Fick-CO in clinical settings and medical researches.

## Author contributions

**Conceptualization:** Sheng-Yi Lin, Feng-Cheng Chang, An-Hsun Chou, Yung-Fong Tsai, Chia-Chih Liao, Chun-Yu Chen.

**Data curation:** Sheng-Yi Lin, Feng-Cheng Chang, Chun-Yu Chen.

**Formal analysis:** Sheng-Yi Lin, Jr-Rung Lin.

**Investigation:** Sheng-Yi Lin, Jr-Rung Lin, Chun-Yu Chen.

**Methodology:** Sheng-Yi Lin, Feng-Cheng Chang, Jr-Rung Lin, An-Hsun Chou, Yung-Fong Tsai, Hsin-I Tsai, Chun-Yu Chen.

**Project administration:** Sheng-Yi Lin, Hsin-I Tsai, Chun-Yu Chen.

**Supervision:** An-Hsun Chou, Chia-Chih Liao, Chun-Yu Chen.

**Visualization:** Sheng-Yi Lin.

**Writing – original draft:** Sheng-Yi Lin.
